# Thyroid hormones modulate irisin concentrations in patients with recently onset hypothyroidism following total thyroidectomy

**DOI:** 10.1007/s40618-020-01432-0

**Published:** 2020-10-14

**Authors:** R. Bocale, A. Barini, A. D‘Amore, M. Boscherin, S. Necozione, A. Barini, G. Desideri, C. P. Lombardi

**Affiliations:** 1 Division of Endocrine Surgery, “Agostino Gemelli” School of Medicine, University Foundation Polyclinic, Catholic University of the Sacred Heart, Rome, Italy; 2 Department of Laboratory Medicine, “Agostino Gemelli” School of Medicine, Institute of Biochemistry and Clinical Biochemistry, University Foundation Polyclinic, Catholic University of the Sacred Heart, Rome, Italy; 3 Department of Life, Health and Environmental Sciences, University of L’Aquila,, Piazza Salvatore Tommasi 1 Coppito, 67100 L’Aquila, Italy

**Keywords:** Irisin, Levothyroxine, Hypothyroidism

## Abstract

**Purpose:**

Irisin is a newly discovered adipo-myokine known for having significant effects on body metabolism. Currently, there is a discussion regarding the relation between thyroid function and irisin concentration. This study was designed to evaluate the influential role of levothyroxine replacement therapy on circulating levels of irisin in patients with recently onset hypothyroidism following total thyroidectomy.

**Methods:**

Circulating levels of thyroid hormones, irisin and other metabolic parameters, were assessed in 40 recently thyroidectomized patients (34 females, mean age 50.1 ± 15.2 years) at baseline (5–7 day after surgery) and after 2 months under replacement therapy with levothyroxine.

**Results:**

At baseline, circulating levels of thyroid hormones were indicative of hypothyroidism (TSH 12.7 ± 5.0 μU/mL, FT3 1.9 ± 0.7 pg/mL, FT4 8.7 ± 3.6 pg/mL). Mean serum irisin concentrations significantly increased after 2 months under replacement therapy with levothyroxine (from 2.2 ± 0.6 to 2.9 ± 0.6 μg/mL, *p* < 0.0001). Variations of circulating levels of irisin under levothyroxine replacement therapy were directly correlated with those of FT3 (Rho = 0.454, *p* = 0.0033) and FT4 (Rho = 0.451, *p* = 0.0035). Multivariate regression analysis revealed that changes in thyroid hormones concentrations explained up to 10% of the variations of serum irisin levels under levothyroxine replacement therapy (FT3 *R*^2^ = 0.098, FT4 *R*^2^ = 0.103).

**Conclusion:**

Our study suggests that levothyroxine replacement therapy mildly influences irisin metabolism in patients with recently onset hypothyroidism following total thyroidectomy.

**Electronic supplementary material:**

The online version of this article (10.1007/s40618-020-01432-0) contains supplementary material, which is available to authorized users.

## Introduction

Irisin, a newly discovered hormone-like adipo-myokine, is the extracellular cleaved product of fibronectin type III domain-containing 5 (FDNC5) and is regulated by peroxisome proliferator-activated receptor gamma (PPARγ) coactivator-1 alpha (PGC1α) [1]. Many researchers have reported its significant influence on metabolism and thermogenesis, mostly by promoting the so-called “browning” of subcutaneous adipose tissues via elevation of uncoupling protein 1, contributing to improvements in glucose homeostasis and insulin resistance [1]. According to this, irisin has been evaluated in different metabolic disorders, such as obesity, insulin resistance, metabolic syndrome, type II diabetes mellitus as well as in subjects with increased cardiometabolic risk [2–4].

Thyroid hormones play important roles in regulating basal metabolic rate and thermogenesis, which may also be affected by irisin. Therefore, thyroid function could directly or indirectly be related to irisin regulation or, vice versa, irisin could affect the thyroid. In this regard, it has been proposed that increased TSH might lead to increased adipogenesis and irisin could be produced to keep the fat distribution in balance in the increased white adipose tissue [5]. Irisin has been studied in patients with thyroid dysfunction with somewhat conflicting results [5–8]. Therefore, it remains to clarify whether the effects of thyroid-axis hormones on metabolism could be possibly mediated by irisin, also because the evidences from interventional human studies that would support a causal effect of thyroid axis on irisin concentrations are currently limited.

This study aimed to evaluate the influential role of replacement therapy with levothyroxine (L-T4) on circulating levels of irisin in patients with recently onset hypothyroidism following total thyroidectomy.

## Materials and methods

A total of 40 thyroidectomized patients (34 females and 6 males, mean age 50.1 ± 15.2 years) were randomly selected among those enrolled in a recently published study from our group aiming to compare the effects of replacement therapy with either liquid or tablet formulations of L-T4 on mood states, self-perceived psychological well-being and thyroid hormone profile in patients with recently onset hypothyroidism following total thyroidectomy [9]. Briefly, enrolled patients initiated L-T4 replacement therapy 5–7 days after the total thyroidectomy. The dose of L-T4 was individualized on the basis of the body weight of patients (about 1.6 mcg/kg of body weight per day). All participants were in good clinical conditions without evidence of relevant comorbidities. Indication for thyroidectomy was represented by multinodular goitre with normal thyroid function while patients treated for thyroid malignancy were excluded [9].

### Laboratory evaluations

All patients, at baseline and then at 2 months of follow-up, underwent blood samplings after an overnight fasting period for determination of the circulating levels of thyroid hormones, serum irisin, glucose, insulin and creatinine and lipid profile. TSH, FT3, FT4 and insulin were measured by COBAS 600 (Electrochemiluminescence Technology, Roche Diagnostics, Mannheim, Germany); reference range for TSH was 0.35–3.2 μU/mL; reference range for FT3 was 2.4–4.2 pg/mL; reference range for FT4 was 8.5–16.5 pg/mL. The homeostasis model assessment of insulin resistance (HOMA-IR) index was calculated according to the following formula: fasting serum insulin (mU/L) × fasting plasma glucose (mmol/L)/22.5. Estimated glomerular filtration rate (eGFR) was assessed by Cockcroft–Gault formula. Irisin concentrations were measured with an enzyme-linked immunosorbent assay (ELISA) kit (measurement range: 0.001–5 μg/mL; sensitivity: 0.001 μg/mL; intrassay variability: 4.86% for 0.678 μg/mL and 7.63% for 1.37 μg/mL: interassay variability: 9.6% for 0.532 μg/mL and 8.02% for 1.14 μg/mL) according to the manufacturer’s directions (Biovendor, laboratory medicine, Czech Republic).

### Statistical analysis

Pre–post differences between the assessed variables were analysed with Wilcoxon Rank-Sign Test after reject hypothesis of normality of their distributions with the Shapiro–Wilk test. Spearman’s nonparametric correlation (Rho) was used to evaluate correlations between variables. Coefficient of determination (*R*^2^) was estimated to evaluate the proportion of the variation in the dependent variable explained by univariate regression models. Multivariate regression models were performed to verify the role of potentially confounding variables. The assumption of normality of the distribution of the residuals (the differences between the observations and the estimated values) was evaluated with the Shapiro–Wilk test. A Statistical analysis was performed using SAS 9.4 (SAS 2002–2012 by SAS Institute Inc., Cary, NC, USA).

## Results

Patients were all in good clinical conditions without relevant diseases. General characteristics of the study population are presented in Table 1. Eight patients (20%) were obese (body mass index > 30 kg/m^2^). Circulating levels of thyroid hormones at baseline were indicative of either subclinical or overt hypothyroidism (TSH 12.7 ± 5.0 μU/mL, FT3 1.9 ± 0.7 pg/mL, FT4 8.7 ± 3.6 pg/mL) (Table 1, supplementary figures 1, 2 and 3). The mean serum irisin concentration at baseline was 2.2 ± 0.6 μg/mL (Table 1 and supplementary figure 4).

**Table 1:** General characteristics of study population (*n* = 40 subjects)

	Baseline	2 months	*p* value*
Gender (females/males)	34/6	–	–
Age (years)	50.1 ± 15.2	–	–
BMI (kg/m^2^)	26.5 ± 5.1	26.6 ± 5.1	0.821
SBP (mmHg)	125.7 ± 10.5	126.0 ± 8.5	0.979
DBP (mmHg)	76.9 ± 6.4	77.4 ± 5.7	0.439
TC (mmol/L)	4.7 ± 0.9	4.8 ± 0.8	0.574
LDL-C (mmol/L)	2.8 ± 0.8	2.9 ± 0.6	0.611
HDL-C (mmol/L)	1.3 ± 0.4	1.4 ± 0.4	0.045
TG (mmol/L)	1.3 ± 0.6	1.1 ± 0.4	0.0009
Glucose (mmol/L)	4.6 ± 0.9	5.1 ± 0.6	0.0007
Insulin (mU/L)	10.4 ± 5.1	10.0 ± 5.0	0.543
HOMA-IR	2.2 ± 1.1	2.3 ± 1.3	0.346
TSH (μU/mL)	12.7 ± 5.0	3.7 ± 4.3	< 0.0001
FT3 (pg/mL)	1.9 ± 0.7	2.9 ± 0.5	< 0.0001
FT4 (pg/mL)	8.7 ± 3.6	15.5 ± 2.7	< 0.0001
eGFR (ml/min)	86.7 ± 26.9	82.7 ± 25.4	0.0017
Irisin (μg/mL)	2.2 ± 0.6	2.9 ± 0.6	< 0.0001

*BMI* Body Mass Index, *SBP* Systolic Blood Pressure, DBP Diastolic Blood Pressure, *TC* Total Cholesterol, *LDL-C* Low Density Lipoprotein Cholesterol, *HDL-C* High Density Lipoprotein Cholesterol, *TG* Triglycerides, *HOMA-IR* Homeostasis Model Assessment–Insulin Resistance, *TSH* Thyroid Stimulating Hormone, *FT3* Free T3, *FT4* Free T4, *eGFR* estimated glomerular filtration rate. Plus–minus values are means ± SD

*Wilcoxon Rank-Sign Test

**Fig. 1:**
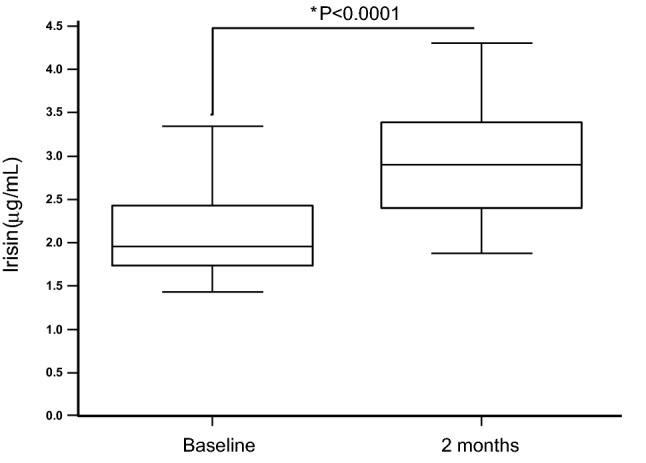
Circulating irisin levels in patients with recently onset post-thyroidectomy hypothyroidism at baseline and after 2 months under replacement therapy with levothyroxine (*p < 0.0001, Wilcoxon Rank-Sign Test)

After 2 months under L-T4 replacement therapy, a significant improvement of thyroid hormone profile was observed in the general study population (Table 1) although a few patients did not achieve a completely satisfactory thyroid hormones replacement or retained the condition of hypothyroidism (supplementary figures 1, 2 and 3). Serum irisin levels significantly increased under L-T4 replacement therapy (Fig. 1 and supplementary figure 4). Circulating levels of both HDL cholesterol and glucose slightly but significantly increased during replacement therapy while triglycerides concentrations significantly decreased (Table 1). A slight but significant reduction of eGFR was observed after 2 months under replacement therapy (Table 1).

Changes in TSH levels after 2 months under L-T4 replacement therapy were significantly correlated with those of FT3 (Rho =  − 0.310, *p* = 0.050) and FT4 (Rho =  − 0.440, *p* = 0.005), while these latter were correlated with each other (Rho = 0.781, *p* < 0.0001).

**Fig. 2:**
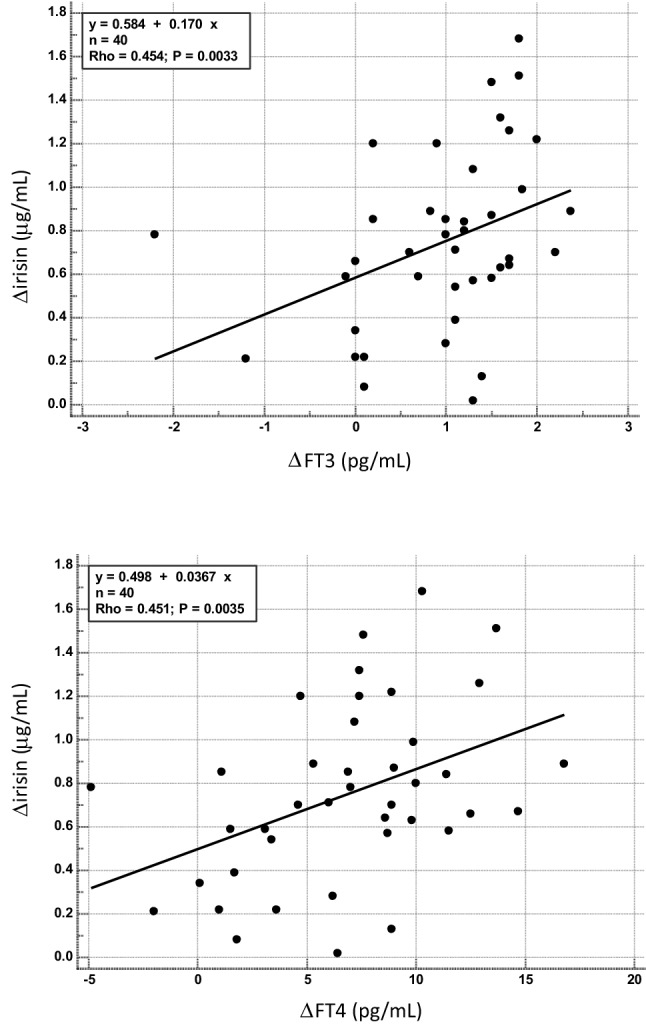
Relationship between changes of irisin levels and changes of FT3 (upper panel) and FT4 (lower panel) in patients with recently onset post-thyroidectomy hypothyroidism after replacement therapy with levothyroxine

Variations of circulating levels of irisin under L-T4 replacement were correlated with those of FT3 (Rho = 0.454, *p* = 0.0033), FT4 (Rho = 0.451, *p* = 0.0035) and glucose levels (Rho = 0.463, *p* = 0.0026), with changes of HOMA-IR (Rho = 0.340, *p* = 0.0317) and eGFR (Rho = − 0.425, *p* = 0.0063) and with age (Rho = − 0.362, *p* = 0.0218). Univariate regression analysis revealed that changes of thyroid hormone levels explained up to 18% of the variations of serum irisin levels under L-T4 replacement therapy (FT3 *R*^2^ = 0.146, *β* = 0.170 *p* = 0.0152; FT4 *R*^2^ = 0.178; *β* = 0.0367 *p* = 0.007) (Fig. 2a, b, respectively). Univariate regression analysis confirmed the relationship of changes of irisin levels with those of glucose (*R*^2^ = 0.110, *β* = 0.146, *p* = 0.037) and eGFR (*R*^2^ = 0.202, *β* = − 0.027, *p* = 0.0036) and with age (*R*^2^ = 0.133, *β* = − 0.010, *p* = 0.0208), but not with those of HOMA (*R*^2^ = 0.076, *β* = 0.0868, *p* = 0.085).

Multivariate regression analysis was performed to verify the role of potentially confounding variables which resulted associated with irisin variations in the Spearman’s correlation analysis. Due to the collinearity between changes of FT3 and FT4, these variables were entered in two distinct models of multivariate analysis. Multivariate regression analysis confirmed the influential role of thyroid hormones on changes of irisin levels under L-T4 replacement therapy (Table 2). Change of eGFR was the only variable, other than thyroid hormones, which remained associated with changes of irisin levels under L-T4 replacement therapy (Table 2).

**Table 2:** Multivariate regression analysis for predicting changes of irisin levels under levothyroxine replacement therapy

	Coefficient	*p*	*R*^2^
Model 1: FT3			
Changes of FT3	0.1468	0.0217	0.098
Changes of eGFR	− 0.02272	0.0181	0.105
Changes of glucose	0.08177	0.1840	0.031
Age	− 0.003670	0.3639	0.014
Shapiro–Wilk test for normal distribution	*W* = 0.9770 accept normality (*p* = 0.5781)		
Model 2: FT4			
Changes of FT4	0.02909	0.0187	0.103
Changes of eGFR	− 0.02119	0.0252	0.092
Changes of glucose	0.09761	0.1047	0.047
Age	− 0.002843	0.4857	0.008
Shapiro–Wilk test for normal distribution	*W* = 0.9691 accept normality (*p* = 0.3361)		

*eGFR* estimated Glomerular Filtration Rate

## Discussion

The main interesting finding of our study was the significant increase of serum irisin levels under replacement therapy with L-T4 in patients with recently onset hypothyroidism following total thyroidectomy.

To date, the available data about the relationship between thyroid function and irisin are scarce and somewhat conflicting. In patients with overt hypothyroidism, circulating irisin levels have been described as either increased [5] or decreased [6, 8]. Similar discrepancies have been found also among studies which evaluate irisin levels in patient with subclinical hypothyroidism. Uc et al. [6] demonstrated that serum irisin levels are lower in patients with Hashimoto’s thyroiditis in comparison to healthy subject and significantly increase following treatment to euthyroid state. More recently, Stratigou et al. [10] described significantly higher circulating irisin levels in patients with subclinical hypothyroidism which were not reverse by 6 months under L-T4 therapy. It was suggested that this might be attributed to the small number of treated patients, the short follow-up period and the variation of irisin not depending solely on TSH levels. On the other hand, two further studies described similar irisin levels in patients with subclinical hypothyroidism and in healthy control [7, 11]. In particular, Yasar et al. [11] enrolled patients with newly diagnosed subclinical hypothyroidism due to autoimmune thyroiditis (Hashimoto’s thyroiditis). The authors hypothesized that thyroid tissue-derived FNDC5/irisin [12] is released in the circulation as a result of the chronic inflammation of the thyroid [11]. On the other hand, Panagiotou et al. [7] evaluated the relationship between thyroid hormonal status and plasma irisin levels in cohort of thyroidectomized patients similar to those enrolled in our study. They found that irisin concentrations were not associated with thyroid-axis hormones cross-sectionally in either the overall cohort of thyroidectomized patients or in the euthyroid and/or subclinical hyperthyroid subgroups. The transient state of hypothyroidism due to levothyroxine withdrawal in patients with thyroid cancer was not associated with a significant change of circulating irisin concentrations. Finally, recombinant human TSH stimulation did not induce any significant changes in circulating irisin levels. Taken together, these findings seem to exclude any effect of TSH on irisin metabolism. On the other hand, the lack of data on the effect of replacement therapy on circulating levels of irisin does not allow to speculate on the possible influential role of thyroid hormones on irisin levels.

Our study sheds new light on this new research field providing a convincing evidence of an influential role of thyroid hormones on irisin metabolism. Indeed, we observed a significant increase of irisin levels under L-T4 treatment which were directly correlated with the increase of both FT3 and FT4 levels. The evidence of a direct relationship between changes of serum irisin levels and those of both FT3 and FT4 seems to suggest the ability of these hormones to modulate irisin metabolism even in the short-term. Since our patients were all euthyroid before thyroidectomy, the present results clearly suggest that the dynamic of the influential role of thyroid hormones on irisin metabolism is quite rapid. In this regard, previous evidence suggested that only long-lasting hypothyroidism is associated with a significant decreases of irisin, possibly as a result from muscle damage due to prolonged myopathy and leakage of irisin from damaged muscle cells, while short-term dysfunction was not associated with changes of irisin concentrations [8].

Our findings seem to be quite robust since they have been obtained in an homogeneous group of patients with recently onset post-thyroidectomy hypothyroidism, selected for having no concomitant clinical conditions potentially influencing irisin levels, including diabetes or chronic kidney disease [13]. Some discrepancies with the previous quoted studies could reflect differences in the enrolled populations, study design and methodological approach.

Although it may be premature, at this point, to conclude on the exact pathophysiological relationship between irisin and thyroid hormones, it is conceivable that irisin levels change, at least in part, according to the thyrometabolic status.

We also found that variations of circulating levels of irisin under L-T4 replacement were directly correlated with those of glucose. Although a positive relationship between serum irisin levels and metabolic risk factors has been already described in sedentary subjects [14], suggesting that metabolic factors, such as glucose or fatty acids, might represent important modulators of circulating irisin levels [15], the entity of this correlation is too small to speculate about an influential role of irisin on glucose metabolism, or vice versa, in our study population. According to this data interpretation, multivariate analysis did not reveal any significant influence of glucose levels on irisin metabolism.

Some limitations of the study should be mentioned. First, because of the study design [9], we cannot establish the circulating levels of irisin before thyroidectomy and thus, we cannot clarify the impact of surgical treatment itself on circulating levels of irisin. In addition, for obvious ethical reasons, our study did not consider a placebo arm in thyroidectomized patients, and thus we cannot completely exclude that a reduction in circulating levels of irisin would have occurred independently on replacement therapy. Second, although all patients underwent total thyroidectomy, we cannot exclude the persistence of some residual thyroid tissue which could have influenced the therapeutic dose required in post-surgical treatment [16, 17]. Anyway, this potential bias likely did not significantly influence our findings since we did not consider the individual L-T4 doses but only the variation of thyroid hormones concentrations under L-T4 replacement therapy. Third, a few patients did not achieve a completely satisfactory thyroid hormones replacement or retained the condition of hypothyroidism, likely because of incompletely adequate adherence to the prescribed therapy. Anyway, this finding likely did not influence our data interpretation since we analyzed the relationship between individual changes of thyroid hormones and irisin concentrations independently on the achievement of an euthyroid status. Finally, given the current controversy around irisin assays and detection methods [18, 19], our finding allows us only to speculate about the pathophysiological relationship between thyroid hormones and irisin metabolism without at this moment clear and immediate clinical implications.

In conclusion, our results demonstrate that L-T4 replacement therapy mildly influences irisin metabolism in patients with recently onset hypothyroidism following total thyroidectomy.

## Electronic supplementary material

Below is the link to the electronic supplementary material.

Supplementary file1 (PPTX 153 kb)

Supplementary file2 (PPTX 149 kb)

Supplementary file3 (PPTX 146 kb)

Supplementary file4 (PPTX 154 kb)

## Data Availability

The datasets generated during and/or analysed during the current study are available from the corresponding author on reasonable request.
