# Supplementary material for: Thyroid hormones modulate irisin concentrations in patients with recently onset hypothyroidism following total thyroidectomy
Source: J Endocrinol Invest. 2020 Oct 14;44(7):1407–12. doi: 10.1007/s40618-020-01432-0 (PMC8195891; doi:10.1007/s40618-020-01432-0)
Supplement: Supplementary file 2 — Supplementary file2 (PPTX 149 kb) [file 40618_2020_1432_MOESM2_ESM.pptx]

## Slide 1
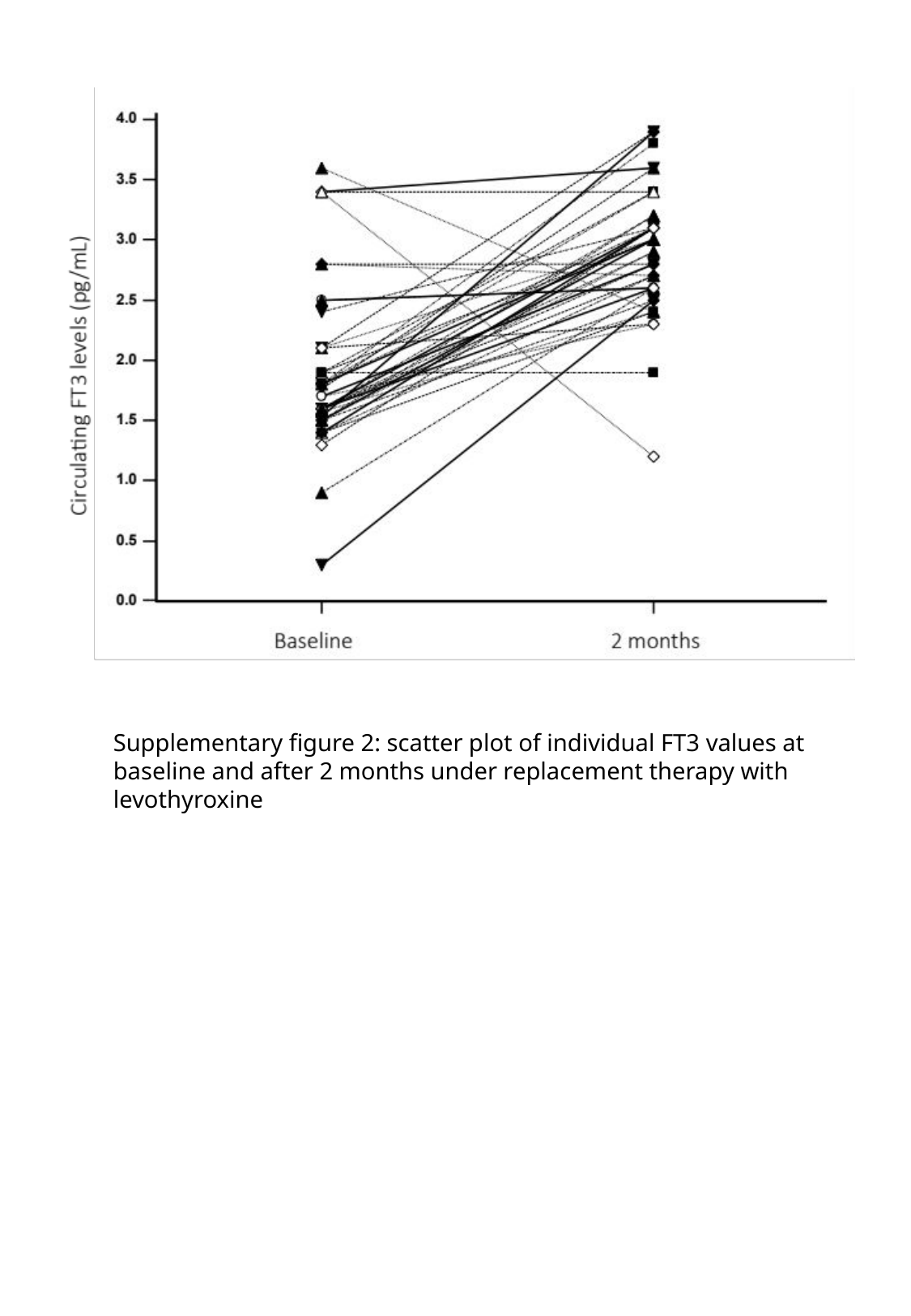

Supplementary figure 2: scatter plot of individual FT3 values at baseline and after 2 months under replacement therapy with levothyroxine
