# Supplementary material for: Thyroid hormones modulate irisin concentrations in patients with recently onset hypothyroidism following total thyroidectomy
Source: J Endocrinol Invest. 2020 Oct 14;44(7):1407–12. doi: 10.1007/s40618-020-01432-0 (PMC8195891; doi:10.1007/s40618-020-01432-0)
Supplement: Supplementary file 3 — Supplementary file3 (PPTX 146 kb) [file 40618_2020_1432_MOESM3_ESM.pptx]

## Slide 1
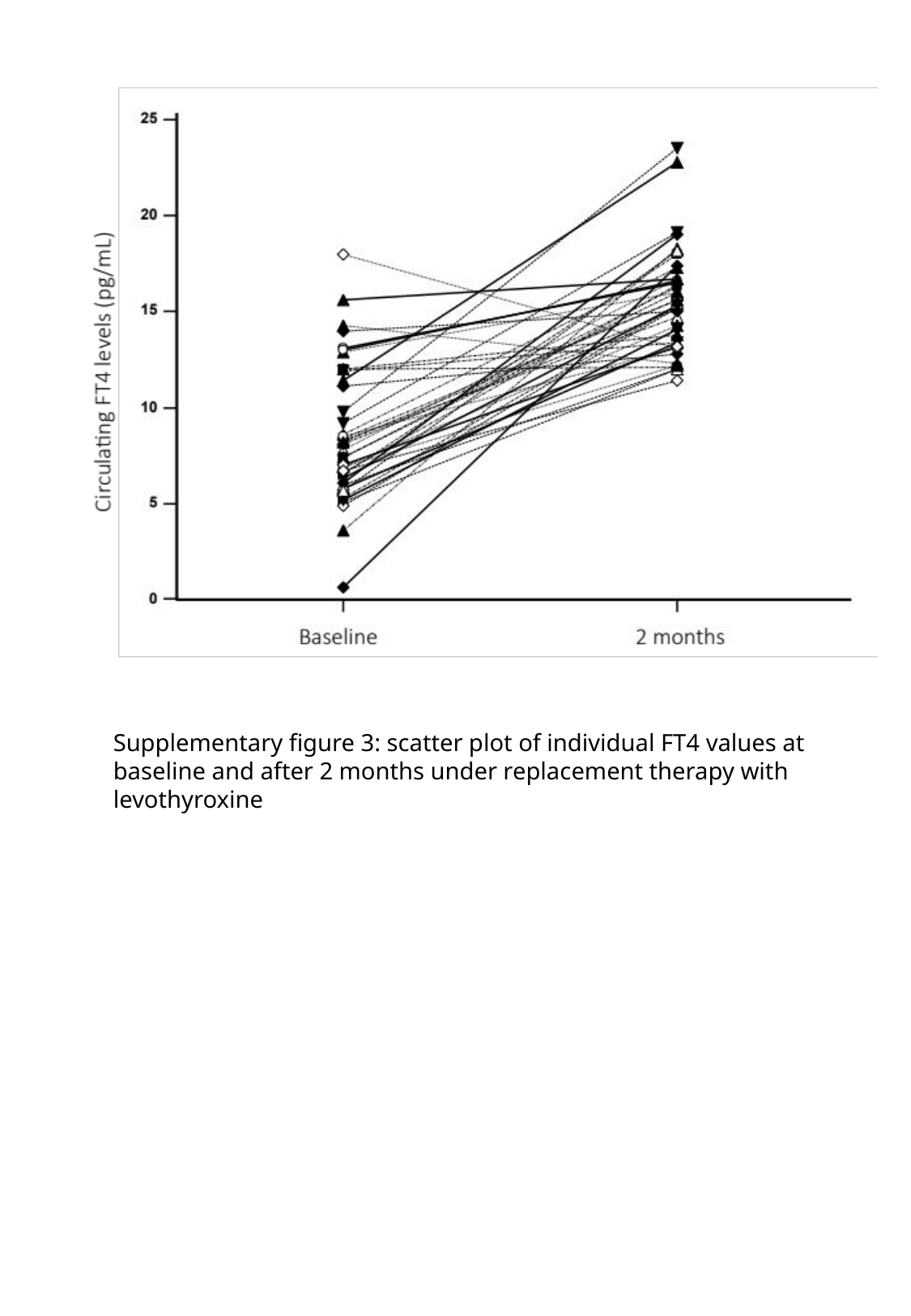

Supplementary figure 3: scatter plot of individual FT4 values at baseline and after 2 months under replacement therapy with levothyroxine
